# Frostbite in Children: A Case Series and Development of a New Iloprost-Driven Protocol: Les engelures chez les enfants : une série de cas et l’élaboration d’un nouveau protocole faisant appel à l’iloprost

**DOI:** 10.1177/22925503261424890

**Published:** 2026-03-10

**Authors:** Kessia S Varkey, Simon J Parsons, Thomas R Cawthorn, Rebecca L Hartley, Yoga Dhanapala, A Robertson Harrop, Frankie O G Fraulin

**Affiliations:** 1 Department of Surgery, Cumming School of Medicine, 2129 University of Calgary, 9978 Alberta Children's Hospital, Calgary, Alberta, Canada; 2 Department of Pediatrics, Cumming School of Medicine, 2129 University of Calgary, 9978 Alberta Children's Hospital, Calgary, Alberta, Canada

**Keywords:** Frostbite, children, iloprost, alteplase, protocol, alteplase, enfants, engelure, iloprost, protocole

## Abstract

**Introduction:** Frostbite in children is uncommon; however, when severe, it can be associated with amputation of digits. Frostbite protocols have been established for adults but not for children. This project outlines our experience with the use of a newly developed iloprost-driven protocol for managing frostbite in children. **Methods:** Motivated by a severe case of frostbite in a teenager, resulting in amputations of multiple digits, the adult Yukon Frostbite Protocol was modified for use in children. Key elements of the new Frostbite Management in Children Protocol include rewarming, iloprost infusion, alteplase administration, and hyperbaric oxygen therapy, if required. Frostbite severity is categorized according to the Cauchy grading system which in turn dictates specific treatment. A review of 3 subsequent cases treated using the protocol was performed. Outcomes recorded included digital amputation rate, motor/sensory recovery, and adverse effects of treatment. **Results:** In December 2022 to March 2024, 3 patients met the criteria for treatment with iloprost under the new protocol: a 9-year-old female with grade 3 frostbite; a 15-year-old male with grade 2 frostbite; and a 16-year-old male with grade 2 frostbite. There were no amputations. All 3 patients recovered motor and sensory function, though one patient experienced significant hypersensitivity. There were no adverse effects from the treatment protocol, and it was well accepted by nursing and medical staff. **Conclusion:** An iloprost-based protocol has been developed for children with frostbite. The protocol was accepted by hospital staff and well tolerated by the patients.

## Résumé

**Introduction :** Les engelures ne sont pas fréquentes chez les enfants, mais lorsqu’elles sont graves, elles peuvent être associées à des amputations des doigts. Il existe des protocoles de traitement des engelures chez les adultes, mais pas chez les enfants. Ce projet expose l’expérience d’utilisation d’un nouveau protocole faisant appel à l’iloprost pour prendre en charge les engelures chez les enfants. **Méthodologie :** En raison d’un grave cas d’engelure chez un adolescent, qui a entraîné l’amputation de plusieurs doigts, le protocole de traitement des engelures du Yukon chez les adultes a été modifié pour être utilisé chez les enfants. Les principaux éléments du nouveau protocole de prise en charge des engelures chez les enfants se composaient du réchauffement, de la perfusion d’iloprost, de l’administration d’alteplase et d’oxygénothérapie hyperbare, si la situation l’indiquait. La gravité des engelures était classée d’après le système de cotation de Cauchy qui, à son tour, dictait des traitements particuliers. Les chercheurs ont procédé à une analyse de trois cas subséquents à l’aide du protocole, qui ont été traités par la suite. Les résultats cliniques incluaient le taux d’amputation des doigts, le rétablissement moteur ou sensoriel et les effets indésirables du traitement. **Résultats :** De décembre 2022 à mars 2024, trois patients ont respecté les critères de traitement faisant appel à l’iloprost en vertu du nouveau protocole : une fillette de neuf ans ayant des engelures de grade 3, un adolescent de 15 ans ayant des engelures de grade 2 et un adolescent de 16 ans ayant des engelures de grade 2. Aucune amputation n’a dû être exécutée. Les trois patients ont récupéré leur fonction motrice et sensorielle, mais l’un deux éprouvait une hypersensibilité marquée. Le protocole thérapeutique n’était associé à aucun effet indésirable et a bien été accepté par le personnel infirmier et médical. **Conclusion :** Un protocole faisant appel à l’iloprost a été mis au point pour les enfants victimes d’engelures, à la fois accepté par le personnel hospitalier et bien toléré par les patients.

## Introduction

Exposure to freezing temperatures, as experienced in northern climates and extreme altitudes, predisposes people to frostbite injury. Additional social factors including homelessness, outdoor work conditions, inadequate clothing, smoking, alcohol and drug consumption increase frostbite risk.^1^ Superficial frostbite heals, while deeper injuries may result in distal limb necrosis.^2^ Deep injuries generally require debridement or amputation followed by reconstruction.^2^ Recently, multimodal treatment protocols have been developed for the treatment of frostbite in the adult population to maximize tissue preservation.^3^ Adult protocols include the Yukon Protocol, the Helsinki Protocol, the K2 base camp Protocol and the Colorado Protocol (Table 1).^3–7^ Most protocols use the clinical grading system described by Cauchy et al in 2016 to estimate the severity of frostbite injuries and to guide treatment.^6^ Two key medications are used in severe frostbite: tissue plasminogen activator (tPA) and Iloprost. Tissue plasminogen activator is a thrombolytic administered within 24 h of rewarming. It binds to fibrin, activating plasminogen and lysing microvascular thrombosis.^8–10^ Iloprost is a synthetic prostacyclin analogue with vasodilatory effects that has been used for severe frostbite cases within 48 h of rewarming.^11^ First described in 1994 in Austria, it became available in Canada through the Health Canada Special Access Program in 2015.^9^ The Yukon protocol for adults recommends using iloprost for grades 2 to 4 frostbite, with a combination of iloprost and tPA for grade 4 treatment.^3,5^ The Helsinki Protocol, K2 base camp Protocol, and Colorado Protocol focus mainly on tPA for frostbite injury due to limited access to Iloprost.^4,6,7^ However, the FDA approved the use of Iloprost for frostbite in the United States in 2024.^12^

**Table 1.** Summary of Current Adult Severe Frostbite Protocol's Treatment Recommendations.

Cauchy Grade	Yukon Protocol	Helsinki Protocol	K2 Base Camp Protocol	Colorado Protocol
Grade 1	Hydrotherapy daily - Aspiration and debridement of clear blisters - Application of silicone dressing and aloe vera - Ibuprofen every 6 h - Elevation of affected area	-Analgesia +/- opioids -Ibuprofen 600 mg oral 3 times daily - No smoking - Limb elevation - Debridement of blisters	N/A	N/A
Grade 2	- Grade 1 management - If within 72 h since rewarming: iloprost intravenous infusion for 5 days, 6 h daily		Prostacyclin (Iloprost) infusion has less risk and used within 48 h of rewarming	N/A
Grade 3		- Angiography if within 48 h from injury and no contraindications If abnormal angiogram - initial papaverine infusion - tPA intra-arterial fibrinolysis and heparin infusion If contraindication with fibrinolysis or vasospasm without angiographic signs of thrombosis or poor response to tPA: - iloprost intravenous infusion for 2-3 days for 6 h		- if the patient presents close to 24 h and no contraindication tPA is given. - tPA administered with loading does of 0.15 mg/kg followed by 0.15 mg/kg/h for 6 h. - immediately after tPA lovenox 1 mg/kg is given and repeated every 12 h for 7 days
Grade 4	- Grade 2/3 management and - intravenous alteplase infusion once - intravenous infusion of heparin adjusted to prothrombin time for 72 h		- Grade 2/3 management and - tPA 1.4 mg/kg over 15 min, if within 24 h from injury along with heparin 1000 U/h for 4 h	

**Figure 1.**
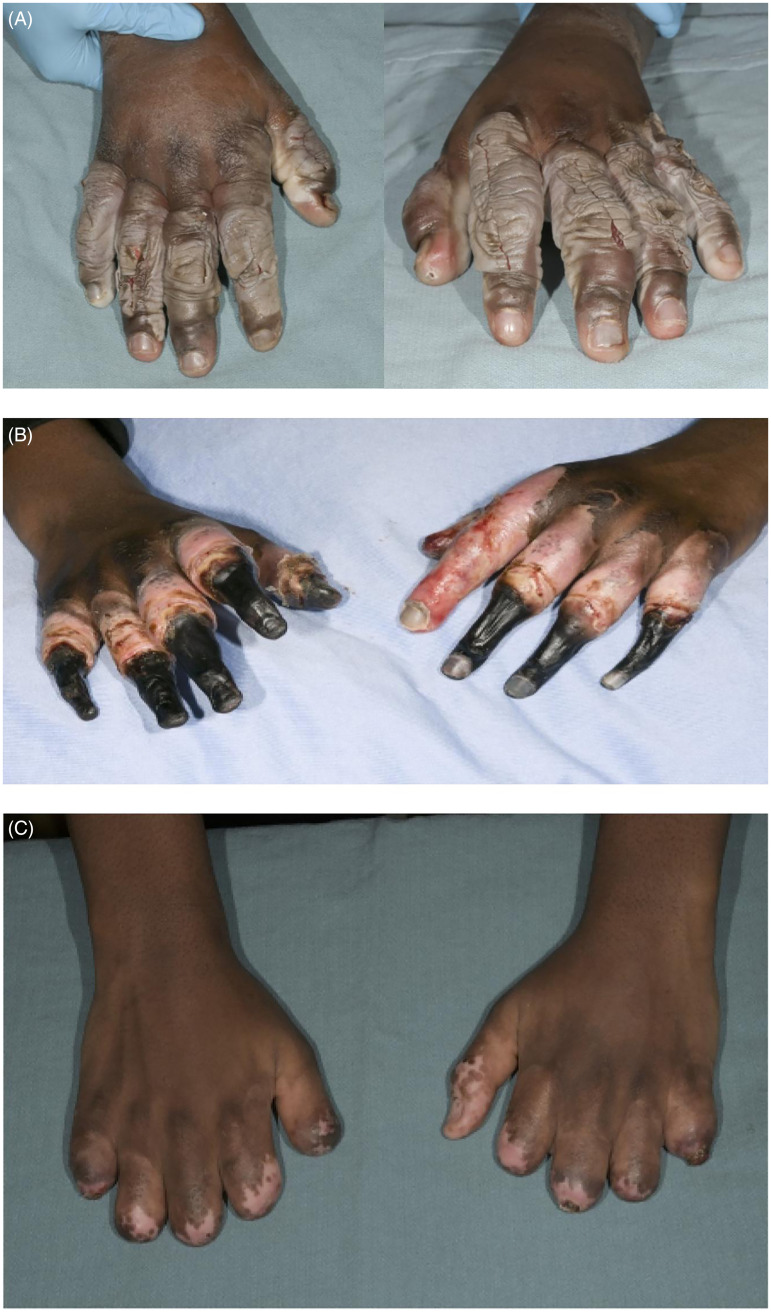
(A) 48 h postinjury; (B) 22 days postinjury; (C) 81 days postinjury.

In 2022, our center treated a severe case of frostbite using elements of the Yukon protocol.^5^ This was the first time iloprost was used in our center. The patient was a 16-year-old male, who was high on lysergic acid diethylamide and alcohol and lost consciousness outside and was exposed to −25 °C temperature for approximately 4.5 h. He had grade 3 frostbite according to the Cauchy grading scale and was treated with an iloprost infusion at 8 µg/h, 6 h/d for 5 days. Despite the treatment, he still went on to develop gangrene in all fingers, requiring surgical amputation (Figure 1).

This case made us reconsider how children are treated for severe frostbite. It was quite evident that there were no published protocols or guidelines for the management of frostbite in pediatric patients at that time. The purpose of this article is to describe the development of an iloprost-driven protocol for the treatment of frostbite in children as well as reviewing the experience in treating 3 children according to the new protocol.

## Methods

### Protocol Development

**Figure 2.**
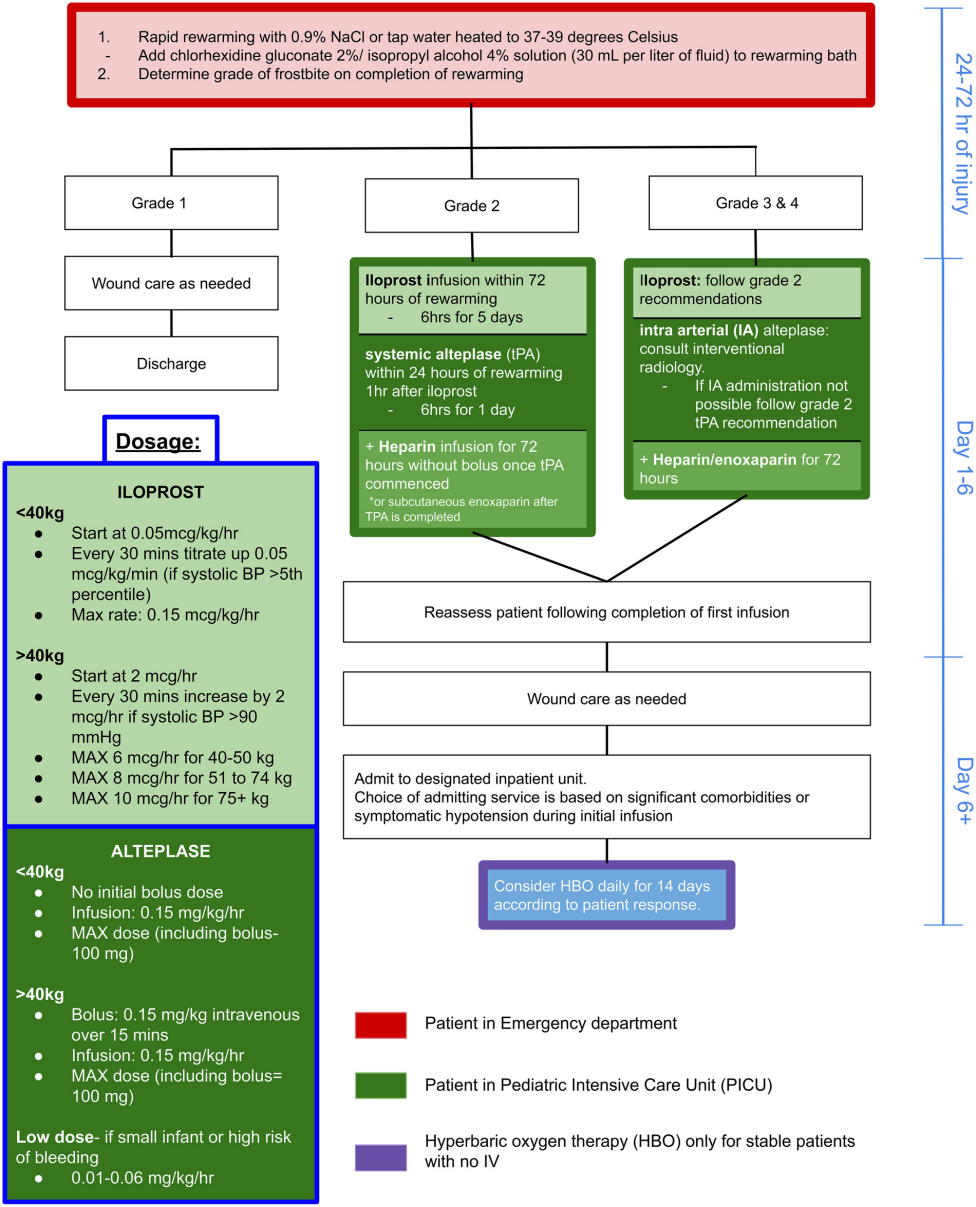
Frostbite management in children protocol.

The Yukon Protocol was the starting point for the development of our pediatric protocol, which we named the Frostbite Management in Children Protocol. The protocol was developed after reviewing all published literature on frostbite and uses a multimodality treatment approach to combat the poor outcomes commonly seen in severe cases (grades 3-4). It includes rewarming, grading of injury, involvement of a multidisciplinary team, specific medical management, and applicability of hyperbaric oxygen (HBO) therapy (Figure 2). There are no published data on the pharmacokinetics of iloprost in children. Due to the lack of pediatric-specific data, iloprost dosages were extrapolated from adult dosing guidelines in our protocol. Iloprost is used for grades 2 to 4 frostbite. Our protocol differs from the adult protocols by suggesting that tPA (Activase rT-PA) with low-molecular-weight heparin be *considered* for grade 2 injuries.^13^ For grades 3 to 4 frostbite, tPA is *recommended* and may be given intraarterially. Exception would be the unavailability of interventional radiology or specifics of the case. Furthermore, HBO therapy is added for grades 3 to 4 injuries or any other case where the response to iloprost and tPA is thought to be inadequate.

This protocol is more aggressive than the adult protocols and overcorrects for the potential of underestimating the frostbite severity. We know that after rewarming, frostbite injury characteristics change through a 24- to 48-h window making it difficult to identify a grade 2 versus 3 or 4 injury. This window is when iloprost and alteplase need to be administered to be effective. Patients commonly present late to the hospital; therefore, rapid aggressive intervention is necessary. A review of 3 subsequent cases treated using the protocol was performed. Outcomes recorded included digital amputation rate, motor/sensory recovery, and adverse effects of treatment.

## Results

The following 3 cases of pediatric frostbite were treated in the Pediatric Intensive Care Unit (PICU) under the new protocol between December 2022 and March 2024.

### Case 1

A 9-year-old female, 33.7 kg, with Fitzpatrick skin type V,^14^ was exposed to −29 °C temperature for approximately 10 min on her walk home from the bus stop without any gloves. Her fingers were rewarmed at home under hot water. The next morning, discoloration and blister formation appeared on her left middle finger. She presented to our center where her left middle finger was cyanotic to the level of the proximal phalanx and determined to have grade 3 frostbite.

**Figure 3.**
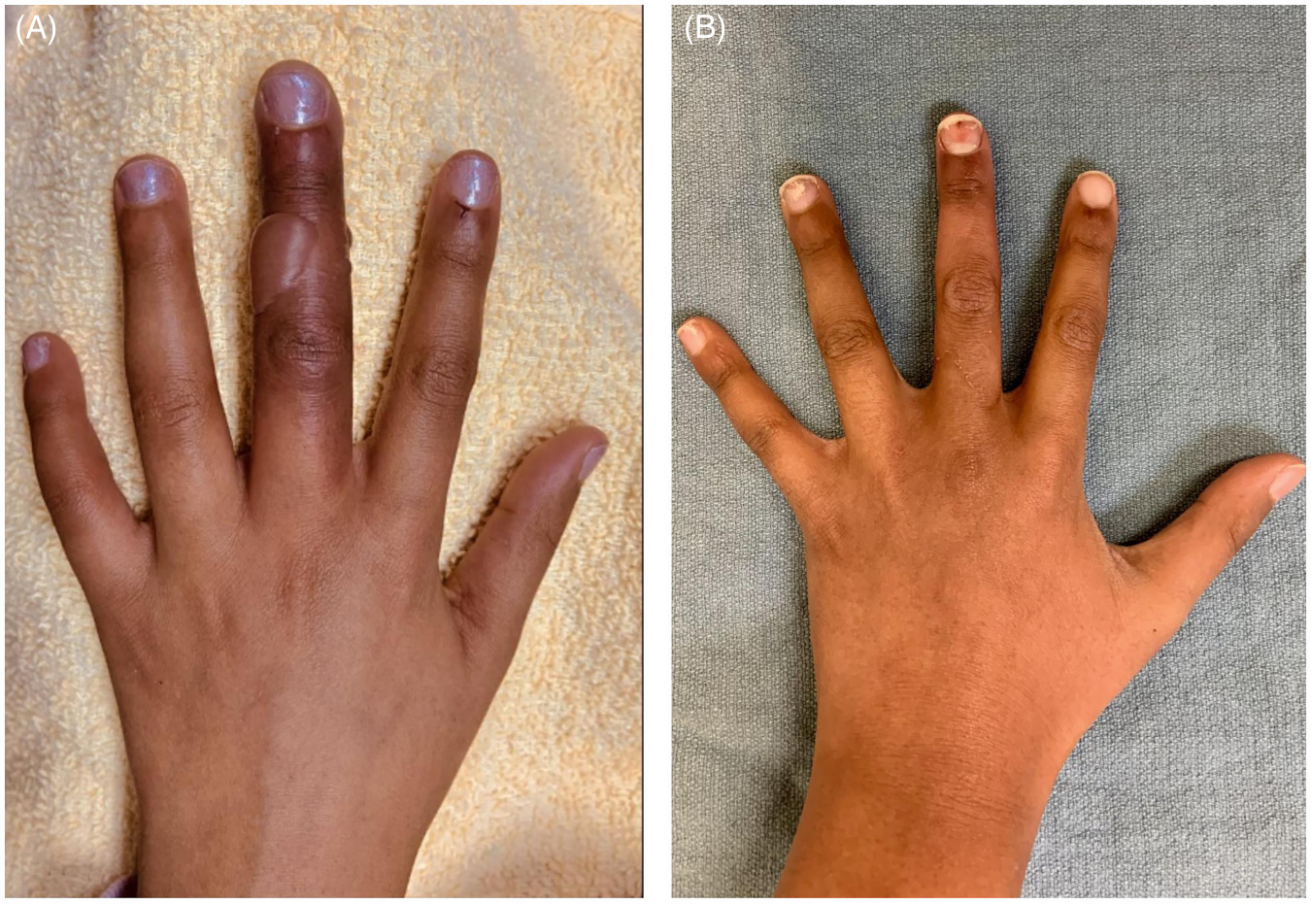
(A) Patient 1: Hand injury Frostbite of middle finger, (B) Patient 1 after treatment and recovery.

Given that the patient presented within 72 h from rewarming but more than 24 h, the protocol called for the administration of iloprost but not tPA. An iloprost infusion was started at a dose of 0.05 µg/kg/h and titrated up to the maximum dose of 0.15 µg/kg/h over 2 h. It was well tolerated, and the patient was transferred to the ward from the PICU. On day 2 of iloprost treatment, there was still poor perfusion to the tip of the left middle finger. The treatment plan was reconsidered. Despite the patient being outside the 24-h postrewarming window, a one-time dose of tPA was administered as an infusion over 6 h at 0.05 mg/kg/h. The patient was started on enoxaparin 72 h after tPA at 1 mg/kg every 12 h. Iloprost was also administered for 6 h each day for the 5 consecutive days. Hyperbaric Oxygen chamber therapy was considered but never initiated, as the closest available HBO chamber was fully booked. By day 5 of iloprost treatment, there was an improvement in warmth and color of the fingertip with no new blisters. At 14 days postinjury, the patient had full recovery with good perfusion, full range of motion and normal sensation (Figure 3).

### Case 2

**Figure 4.**
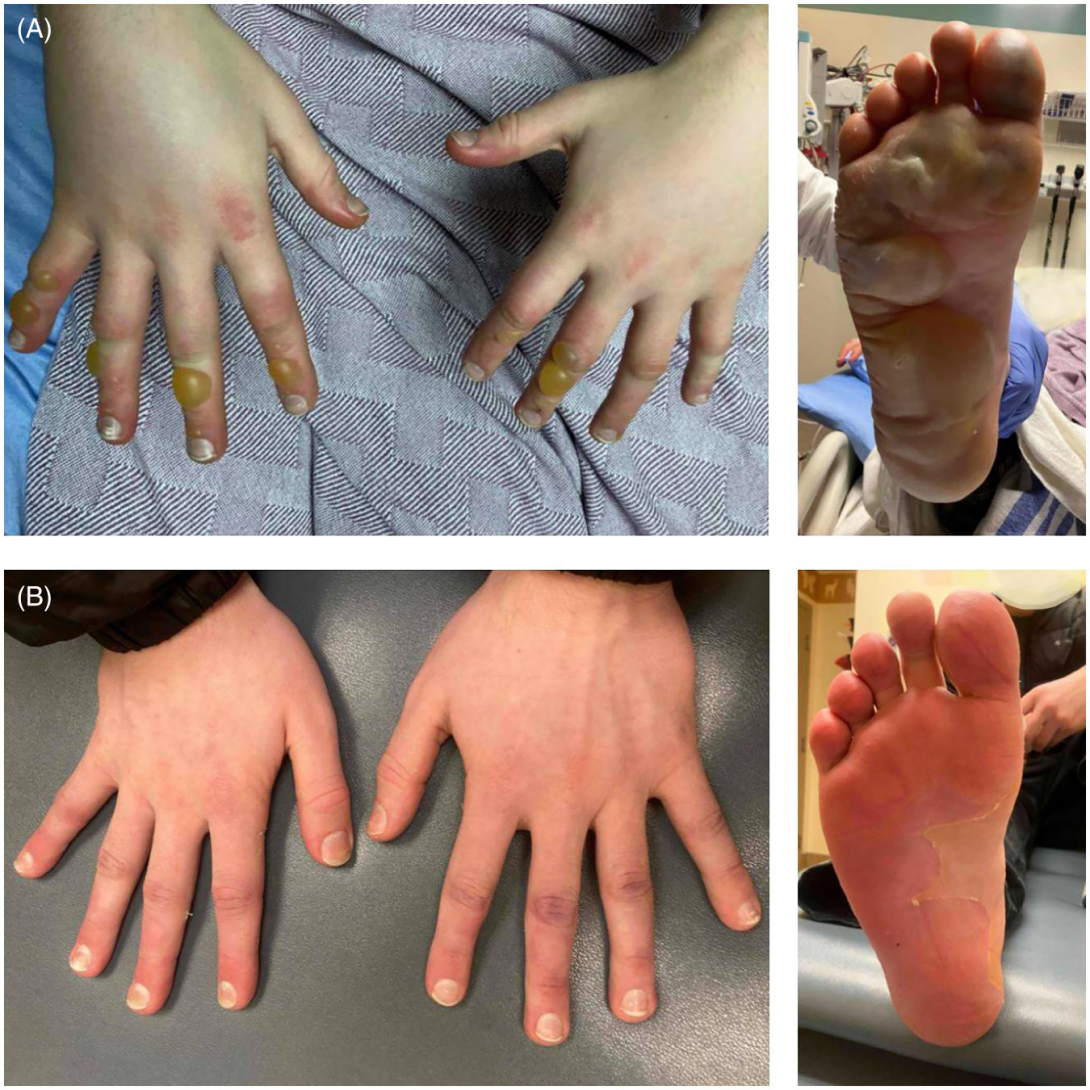
(A) Patient 2: Hand and Foot Frostbite within 24 h of injury showing signs of swelling, blistering, and discoloration. (B) Patient 2, 28 days postinjury, showing full recovery.

A 15-year-old male, 79.1 kg, with Fitzpatrick skin type II was drinking alcohol, using marijuana and mushrooms with friends outside in January only wearing light gloves and one boot and was exposed to −35 °C for 1.5 h. He rewarmed at home with many blankets. He presented to the emergency department 36 h after the injury, with cyanosis on the tips of the fingers, blistering, and swelling of all digits on the right hand, as well as the index and middle fingers of the left hand. The right foot was also swollen with large blisters over the plantar surface. The frostbite was determined to be grade 2 (Figure 4).

Iloprost was administered at 2 µg/h titrated up to 10 µg/h and administered for 6 h daily for 5 days. After the first dose of iloprost, his cyanosis improved. He received bedside blister debridement and wound dressings for his feet and hands. The patient was discharged on day 6 after the full course of iloprost treatment. He was seen again at 31 days postinjury, and all areas affected by the frostbite had completely recovered (Figure 4).

### Case 3

A 16-year-old male, 75.1 kg, with Fitzpatrick skin type V, was shovelling snow in March. Though wearing gloves, he was exposed to −21 °C temperatures for approximately 1 h before rewarming his hands. He presented to the emergency department 3.5 h after rewarming where he was noted to have discoloration, swelling, and blistering on the fingers of both hands. The frostbite was determined to be grade 2.

**Figure 5.**
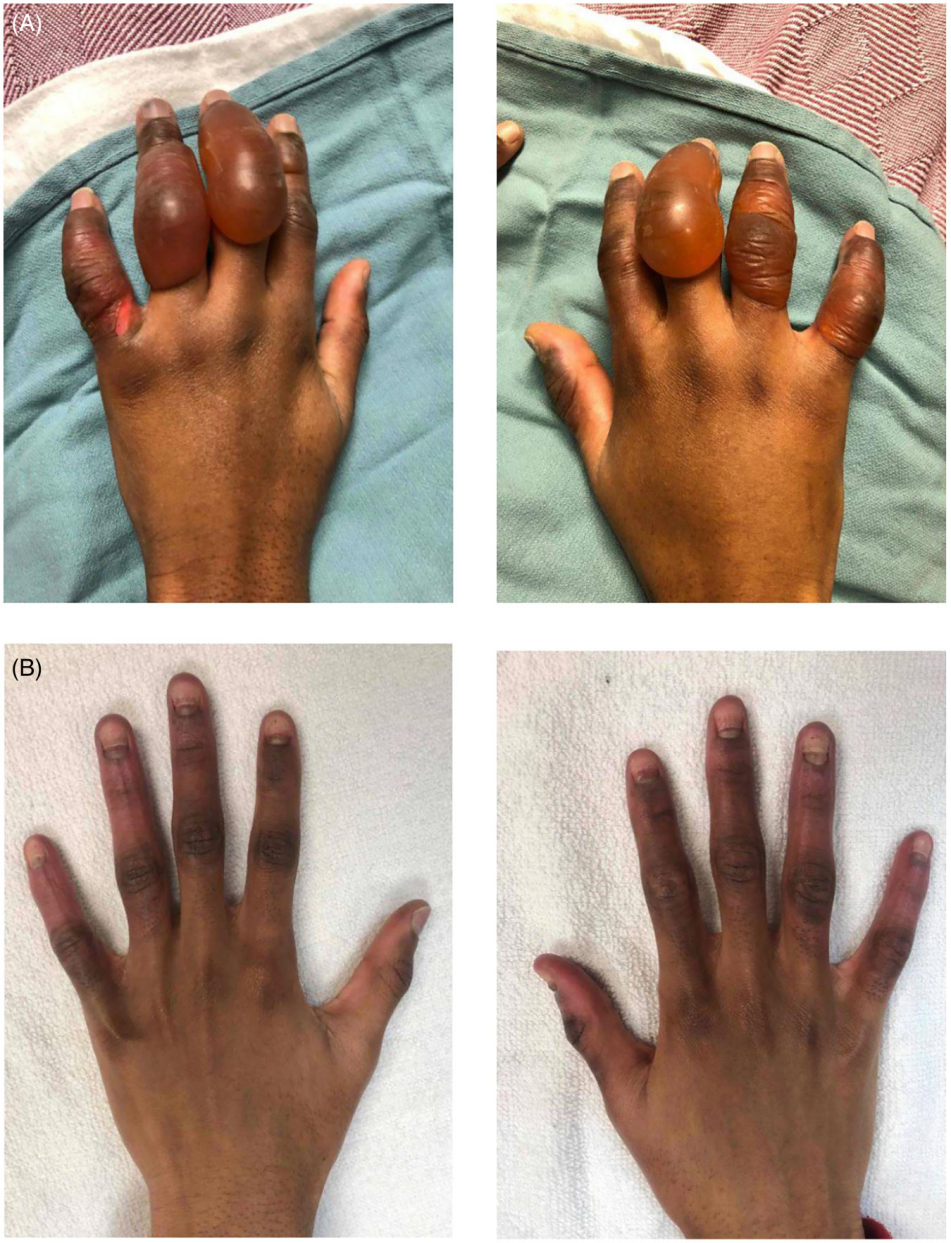
(A) Patient 3: Bilateral hand Frostbite within 24 h of injury showing signs of swelling, blistering, and discoloration on both palmar and dorsum aspect of digits. (B) Patient 3: 46 days postinjury showing signs of hyperpigmentation on all fingers and development of new nails.

An Iloprost infusion was started at 2 µg/h and titrated up to 10 µg/h. Because perfusion was improving with the iloprost, alteplase was not used. This patient experienced pain in all digits, especially the left index finger. Blisters were debrided at the bedside. He was discharged after 8 days. By 46 days postinjury, both hands had healed, though he had lost the nail plates on all digits in both hands except the left thumb. He had good range of motion but still complained of tingling in his fingertips. The altered sensation was noted to have disappeared, and the nail plates to have regrown at 3 months post injury (Figure 5).

The cases are summarized in Table 2. Overall, the protocol was well tolerated by patients with no significant complications. There were no concerns from hospital staff regarding implementation of the new protocol.

**Table 2.** Summary of Frostbite Treatment and Outcome in Paediatric Frostbite.

Case #	Age & Sex	Frostbite Grade / Body Part	Iloprost	LMW Heparin	TPA (Alteplase)	Analgesics	Wound Care	Outcome
1	9 years—female	Grade 3 hands	Infusion started at 1.68 µg/h and increased to a max of 5.04 µg/h (5 days)	Enoxaparin 100 mg/mL injection 34 mg 1 mg/kg/dose, subcutaneous (3 days)	50 mg in 50 mL 0.05 mg/kg/h, IV, continuous (1 day)	Acetaminophen liquid 480 mg PO q6 h PRN	Polysporin and Bandages Blisters drained	Full ROM Perfusion and sensation normal No tissue loss, no amputation Length of stay: 5 days
2	15 years—male	Grade 2 hands & right foot	Infusion started at 2 µg/h and increased to a max of 10 µg/h (5 days)	NA	NA	Acetaminophen tablet 650 mg, oral, q6 h PRN Ibuprofen tablet 400 mg oral, q6 h PRN Acetylsalicylic acid-81 mg for 4 weeks	Blisters drained & deroofed Wrapped with Mepilex Ag and gauze	Full ROM All areas healed. Some sensitivity on planter surface of right foot. No loss of digits Length of stay: 6 days
3	16 years—male	Grade 2 hands	Infusion started at 2 µg/h and increased to a max of 10 µg/h (5 days)	NA	NA	Ibuprofen 400 mg PO q6 h PRN Ketorolac-30 mg 15 mg q6 h sc Acetaminophen 1000 mg PO q6 h PRN Hydromorphone twice Pregabalin-75 g bid Acetylsalicylic acid-81 mg PO daily	Blisters drained & deroofed	Full ROM Hyperpigmentation on all fingers Loss of all nail plates except left thumb No loss of digits Length of stay: 8 days

## Discussion

Every winter our center sees many children with frostbite. However, severe frostbite to the extent that the digits are at risk for amputation is rare. If we do not include the patient described in the introduction, we have only had 3 significant cases over a span of 15 months (December 2022-March 2024). A review of the pediatric frostbite literature published in 2024 outlined 4 studies specifically targeting pediatric frostbite, exposing a gap in the current available body of research.^15^ Though iloprost is widely used in frostbite in adult patients, there are no reports of iloprost use in children.^16^ The following information represents our recommendation regarding the treatment of frostbite in children.

### Rewarming

Rewarming should be initiated after the risk of further exposure to cold temperatures has passed. Patients should undergo rapid rewarming in normal saline or water at 37 °C to 39 °C.^17^ This typically takes 30 to 60 min. If in a hospital setting 2% chlorhexidine gluconate may be added to the rewarming solution (30 mL per litre of fluid).^17^

### Grading

Grading of the frostbite injury should take place after rewarming is completed. The Cauchy grading scale is based on the proximal extent of cyanosis in the worst affected digit.^6^ Facial frostbite (ie, ears, cheeks, and nose) should also be assessed and considered for infusion therapy, although experience with infusion therapy for such cases is limited. There may be difficulty with the Cauchy grading system in children and in patients with darker skin color. The degree of cyanosis may be worse than is first estimated. We propose that the extent of the blistering, whether clear or hemorrhagic should be taken into consideration. For example, even if cyanosis does not seem to progress to the MCP joints, but blistering does, it might be best to categorize these injuries as grade 3 and be more aggressive with treatment including both iloprost and tPA. We learned about this risk of blistering from the case presented in the introduction. That patient only had clear blisters that extended to the MCP joints, and he went on to develop gangrene and loss of portions of his digits.

### Admission to ICU and Involvement of Multidisciplinary Team

Patients are assessed by emergency room physicians and the plastic surgery service. Those with grades 2, 3, or 4 injuries are considered for iloprost and tPA. They are admitted to the PICU, where a review by the multidisciplinary team (plastic surgery, intensive care staff, hematology, and interventional radiology [if needed]) takes place.

### Iloprost

A 2011 controlled trial indicated the effectiveness of iloprost in minimizing amputation rates in grades 2, 3, and 4 injuries in an adult population.^18^ There are, however, only a few reports of the use of Iloprost in grade 4 injuries in children and these involved adolescent-aged patients. Patient history must be carefully evaluated for any contraindications to the use of Iloprost: hypersensitivity to iloprost, pregnancy, high risk of hemorrhage, hepatic injury, recent cerebrovascular event, and heart disease or arrhythmia. If less than 72 h from the time of rewarming, iloprost is indicated as follows:

### Patients Under 40 kg

- Start Iloprost at 0.05 µg/kg/h
- Titrate up every 30 min by 0.05 µg/kg/h to a target dose of 0.15 µg/kg/h
- If hypotension occurs, hold Iloprost at the highest tolerated dose

### Patients Over 40 kg

- Start Iloprost at 2 µg/h, and increase by 2 µg/h every 30 min to a maximum dose of:
  - 6 µg/h (patients 40-50 kg)
  - 8 µg/h (patients 51-74 kg)
  - 10 µg/h (patients 75 kg or more)
- If hypotension occurs, hold Iloprost at the highest tolerated dose

### Continue Infusion for 6 h

#### Repeat Daily for 5 Days, Starting at the minimum Tolerated Dose of the Previous day

Iloprost infusions were well tolerated, successful, and resulted in a maximum infusion rate by day 3 for all patients. Side effects that can occur due to iloprost are flushing, jaw pain, headaches, hypotension, nausea, diarrhea, and dizziness.^19^ Of note, iloprost has significant anticoagulant activity, so there is a risk of bleeding, but this complication is not reported in the literature. The suggestion of 6 h of iloprost infusion per day is also not absolute. If clinical deterioration occurs once iloprost is stopped, prolonging the infusion is acceptable. Iloprost can be run continuously for more than 5 days, as in one study it was used continuously for 8 days.^18^

#### Tissue Plasminogen Activator

For cases at high risk of amputation, tPA can be used as a thrombolytic within 24 h after rewarming.^15^ Alteplase is a type of tPA commonly used for thrombolysis in frostbite. Studies show that additional therapies alongside iloprost can increase the likelihood of a no-digit amputation outcome and increase tissue salvage.^9,10,15^ Based on our institutional experience and the adult frostbite literature, our protocol recommends considering Iloprost in all pediatric patients with grades 2 to 4 frostbite. Under our protocol, alteplase is *considered* in addition to iloprost for grade 2 injuries and *recommended* for grades 3 and 4. Alteplase dosing is based on weight. Each hour alteplase is delayed there is a decreased salvage rate of 28%.^17^

##### Patients Under 40 kg

- No initial bolus.
- Infusion: 0.15 mg/kg/h for a total of 6 h (max of 100 mg).

##### Patients Over 40 kg

- Bolus 0.15 mg/kg intravenous over 15 min.
- Infusion: 0.15 mg/kg/h for a total of 6 h (max of 100 mg).

Newer case series suggest that direct intra-arterial alteplase may result in higher rates of upper extremity digital salvage than systemic alteplase, thus interventional radiology should be consulted for frostbite grades greater than 2.^20^ Complication rates are similar for intra-arterial and intravenous alteplase protocols, hence the route of administration can be determined on a case-by-case basis. Complications reported from alteplase include bleeding resulting in compartment syndrome, intracranial hemorrhage, hypersensitivity, and angioedema.^21^

### Deviations from Protocol

In Case 1, given the significant risk of amputation, alteplase treatment was supported despite the injury occurring more than 24 h after rewarming. The Yukon protocol and the Colorado protocol both suggest a 0.15 mg/kg loading dose and others recommend a 0.5 mg/kg bolus.^5,7^ These were not deemed to be a suitable option for our pediatric patient, and an alternative approach of low-dose therapy was used—alteplase (0.01-0.06 mg/kg/h). We know that there is a significant, albeit small, risk of bleeding with the use of alteplase in children that may be higher than seen in adults, and as such the optimal pediatric dose is not known. Lower dose regimes may be equivalent in efficacy to higher dose regimens based on case series on nonfrostbite-related arterial thrombosis.^22^ If low-dose alteplase is used, anticoagulation via low-molecular-weight heparin should be continued for 72 h; however, some centers continue for 14 days.

### Pain Management

Adequate pain relief is important from the rewarming period onward. Some cases required narcotics, and our center used hydromorphone; this has been supported in the literature.^27,28^ Acetaminophen can also be taken for managing pain from frostbite injury. Our protocol suggests NSAIDs, for the benefit of the antiplatelet effect, but should be delayed until after alteplase administration is completed. Acetylsalicylic acid can be used for its anti-inflammatory and antiplatelet effects. No published studies compare the effects of these options in frostbite cases.^17^ Gabapentin or pregabalin is helpful for the control of neuropathic pain associated with frostbite.^29^

### Hyperbaric Oxygen Therapy

The Yukon adult frostbite protocol does not include recommendations for HBO therapy. There is some weak evidence that it may have additional benefits in tissue preservation and minimizing amputation rate when added to iloprost as an early or late treatment option.^23,24^ Accelerated healing occurs in many types of wounds due to increased tissue oxygenation from HBO therapy.^25^ We have included HBO therapy recommendation in our pediatric frostbite protocol for grades 2 or greater daily for up to 14 days, if the therapeutic response to iloprost and alteplase is deemed inadequate by day 2. The article by *Magen et al* proposes recommendations for HBO therapy, but these recommendations are not yet supported by large-scale clinical trials. However, we have included it as an adjunctive option based on theoretical benefits.^26^ We recognize that there are practical issues around access to HBO in many centers, including our own.

### Delayed Treatment

Our protocol suggests that patients must present within the first 72 h to be considered for iloprost, but we may not be rigid on that recommendation. Time limit recommendations of less than 72 h for iloprost and less than 24 h for alteplase should be considered suggestions and not absolute. There are case reports of benefit from late treatment and in our series, case 1 is an example of this for late alteplase treatment.^22^ Smaller regional centers do not have Iloprost available. After discussions with our intensive care team, urgent alteplase administration could be recommended before transfer to the hospital for grade 3 or 4 disease. If iloprost and alteplase are not used, HBO should still be considered.

### Future Directions

This case series is intended to be the starting point for research in pediatric frostbite injuries treated with iloprost and alteplase. It describes the journey that led to the creation of the Frostbite Management in Children Protocol and the following cases treated under this protocol. For future studies, we hope to further support the validity of the suggestions presented as more cases are treated under this protocol. Furthermore, there is a frostbite website and registry in Canada organized by Dr Alex Poole that is a useful resource for the public and health care professionals (https://frostbitecare.ca/). With collection of data from multiple pediatric centers, this registry has the potential to help modify and update our protocol, and other protocols, to treat severe frostbite in children.

## Conclusion

An iloprost-based protocol has been developed and utilized in 3 children with frostbite. The protocol was accepted by hospital staff and well tolerated by the patients.

## Supplemental Material

**Figure:** Video 1.
